# Massively Parallel Sequencing of Patients with Intellectual Disability, Congenital Anomalies and/or Autism Spectrum Disorders with a Targeted Gene Panel

**DOI:** 10.1371/journal.pone.0093409

**Published:** 2014-04-01

**Authors:** Maggie Brett, John McPherson, Zhi Jiang Zang, Angeline Lai, Ee-Shien Tan, Ivy Ng, Lai-Choo Ong, Breana Cham, Patrick Tan, Steve Rozen, Ene-Choo Tan

**Affiliations:** 1 KK Research Centre, KK Women's & Children's Hospital, Singapore, Singapore; 2 Duke-NUS Graduate Medical School, Singapore, Singapore; 3 National Cancer Centre, Singapore, Singapore; 4 Genetic Services, KK Women's & Children's Hospital, Singapore, Singapore; 5 Universiti Malaya Medical Centre, Petaling Jaya, Malaysia; The Chinese University of Hong Kong, Hong Kong

## Abstract

Developmental delay and/or intellectual disability (DD/ID) affects 1–3% of all children. At least half of these are thought to have a genetic etiology. Recent studies have shown that massively parallel sequencing (MPS) using a targeted gene panel is particularly suited for diagnostic testing for genetically heterogeneous conditions. We report on our experiences with using massively parallel sequencing of a targeted gene panel of 355 genes for investigating the genetic etiology of eight patients with a wide range of phenotypes including DD/ID, congenital anomalies and/or autism spectrum disorder. Targeted sequence enrichment was performed using the Agilent SureSelect Target Enrichment Kit and sequenced on the Illumina HiSeq2000 using paired-end reads. For all eight patients, 81–84% of the targeted regions achieved read depths of at least 20×, with average read depths overlapping targets ranging from 322× to 798×. Causative variants were successfully identified in two of the eight patients: a nonsense mutation in the *ATRX* gene and a canonical splice site mutation in the *L1CAM* gene. In a third patient, a canonical splice site variant in the *USP9X* gene could likely explain all or some of her clinical phenotypes. These results confirm the value of targeted MPS for investigating DD/ID in children for diagnostic purposes. However, targeted gene MPS was less likely to provide a genetic diagnosis for children whose phenotype includes autism.

## Introduction

Developmental delay and/or intellectual disability (DD/ID) affects 1–3% of all children and at least half of these are thought to have a genetic etiology. The genetic causes include microscopically visible chromosomal imbalances, copy number variants as well as point mutations. However, the genetic etiology remains unknown for at least 50% of all cases of DD/ID [1]. Diagnosis of DD/ID is challenging due to the broad spectrum of phenotypic presentations, as patients with DD/ID often have congenital anomalies and/or autism spectrum disorders. With the advent of massively parallel sequencing (MPS) or next generation sequencing (NGS), there has been expectation of an increased detection of the genetic causes of DD/ID. In particular, exome sequencing has been identified as an effective tool for discovery of new disease genes. However, many technical challenges remain including the uneven depth of coverage across the exome, gaps in coverage and mapping problems. In addition, there is the challenge of interpreting the clinical significance of the thousands of variants generated by exome sequencing.

An alternative approach is to perform MPS with a targeted gene panel. Studies using panels of targeted genes have been reported for various disease entities like cardiomyopathies [2], hearing loss [3], epilepsy [4] and retinal disorders [5]. These studies have shown that targeted MPS is particularly suited for clinical diagnostic testing for genetically heterogeneous conditions where there are a large number of known candidate genes.

We describe our experiences with using massively parallel sequencing of a targeted gene panel of 355 genes for investigating the genetic etiology of eight patients with a range of phenotypes including DD/ID, congenital anomalies and autism spectrum disorder.

## Materials and Methods

### Ethics Statement

This study is approved by the Institutional Review Board of KK Women's and Children's Hospital and SingHealth Centralised Institutional Review Board A. The patients were recruited with written informed parental consent by the Genetics Service, KK Women's & Children's Hospital, Singapore.

### Patient phenotypes

Eight patients with phenotypes ranging from developmental delay, multiple congenital anomalies and/or autism spectrum disorders were selected for the targeted sequencing (Table 1). Prior genetic tests were normal and array comparative genomic hybridization did not detect any copy number changes associated with known microdeletion and microduplication syndromes.

**Table 1:** Phenotypes and genetic testing of the eight patients.

Patient	Age	Gender	Phenotypes	Previous genetic testing
Patient 1	11	Male	DD/ID, hydrocephalus, adducted thumbs, agenesis of corpus callosum, spasticity, optic atrophy, CTEV, no speech	Karyotype, aCGH
Patient 2	4	Male	DD, microcephaly, dysmorphism (hypertelorism, low set ears, posteriorly rotated, microstomia with tented upper lip, high forehead with cowlick), hypotonia, short fingers, bilateral CTEV, bifid scrotum, undescended testes, speech delay, family history of neurodevelopmental disorders and early deaths	aCGH
Patient 3	4	Female	Dandy-Walker malformation, bilateral post-axial polydactyly, ventricular septal defect; anal stenosis, hearing loss, omphalocoele minor, hypoplastic nipple, sacral dimple, low set ears, deep set eyes, significant tendency to keloid formation	Karyotype, fluorescent in-situ hybridization for 6p deletion, aCGH
Patient 4	11	Female	DD, autism spectrum disorder diagnosed at age 3½.	Karyotype, Angelman Syndrome, aCGH
Patient 5	5	Male	DD, no speech, no eye contact	Karyotype, FragX, aCGH
Patient 6	5	Male	Speech delay, autism spectrum disorder, sister with DD and Turner's syndrome	Karyotype, FragX, aCGH
Patient 7	11	Male	Normal IQ, mild autism, does not interact with peers, hyperactive	Karyotype, aCGH
Patient 8	7	Female	DD, intellectual disability? autism spectrum disorder, speech delay, moderate IQ, hypertelorism, depressed nasal bridge, prominent jaw, brother with ADHD	Karyotype, FragX, aCGH,

DD  =  developmental delay; ID  =  intellectual disability; CTEV  =  congenital talipes equinovarus; ADHD  =  attention deficit hyperactivity disorder; aCGH  =  array comparative genomic hybridization; FragX  =  Fragile X

### DNA extraction

Genomic DNA was isolated from peripheral blood using the Gentra Puregene blood kit (Qiagen Inc.,USA) according to the manufacturer's instructions.

### Targeted gene panel and MPS

A total of 355 genes were targeted for capture and deep sequencing. They include candidate genes associated with DD/ID, microdeletion/microduplication syndromes, congenital anomalies, and autism spectrum disorders (Table S1). Using the eArray system (Agilent Technologies Inc. USA), the capture was designed to include exons with at least 30 bp of the flanking intronic sequence. In addition, 5 kb of the flanking sequence in the 5′UTR and 3′UTR regions were added to the targeted regions. A total of 4150 regions from the 355 genes were targeted for a final capture size of 4.79 Mb.

Targeted sequence enrichment was performed using the Agilent SureSelect Target Enrichment Kit (Agilent Technologies Inc. USA). Genomic DNA was sheared using a Covaris S1 Ultrasonicator (Covaris, MA). Adaptor-ligated libraries were constructed using Paired-End Genomic DNA kits (Illumina, CA). The multiplexed samples were sequenced on the Illumina Hiseq platform using 76-bp paired-end reads.

### Variant analysis and prioritization

Sequencing data were aligned to hg19 using the Burrows-Wheeler Aligner (BWA) software. PCR duplicates were removed with the ‘samtools’ software, and variants called using the Genome Analysis Toolkit (GATK, version 1.0) software from the Broad Institute.

**Figure 1:**
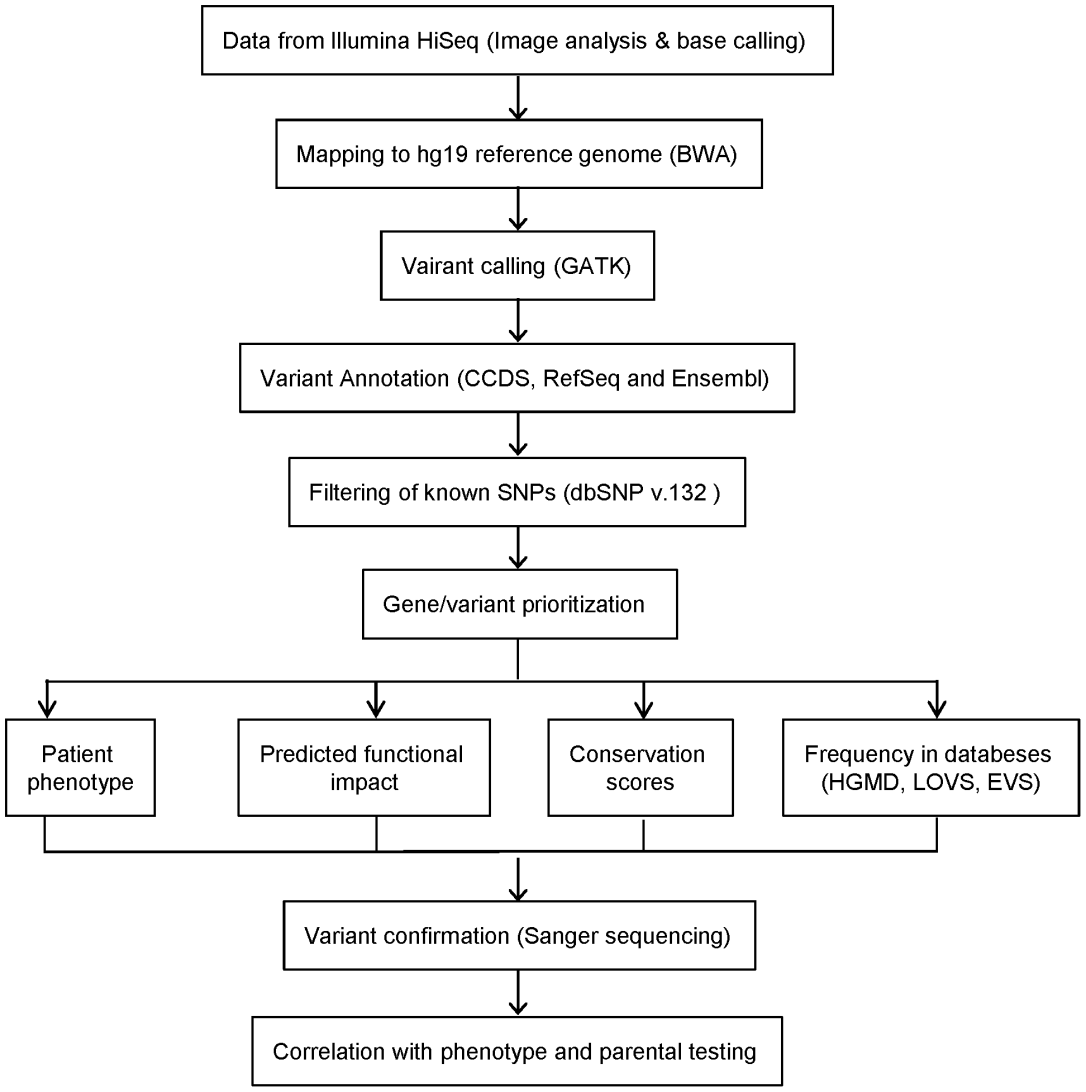
Variant analysis and prioritization workflow. Summary of our variant evaluation process for identifying candidate mutations

Variants were annotated based on CCDS, Refseq and Ensembl gene transcripts. Identified variants that are listed in the NCBI dbSNP (version 132) were filtered out, along with variants that had poor depth or low average base quality scores. Prioritization of variants was carried out by the following criteria: selection of candidate genes based on patient's phenotype, severity of the predicted impact on gene function, conservation of amino acid affected, and frequency of the variant in the literature and databases including the Human Gene Mutation Database (HGMD http://www.biobase-international.com/), Leiden Open Variation Databases (LOVD http://www.lovd.nl/3.0/home), Exome Variant Server (EVS, http://evs.gs.washington.edu/EVS/) and 1000 genomes database (http://browser.http://browser.1000genomes.org/index.html). PolyPhen-2 (http://genetics.bwh.harvard.edu/pph2/) and SIFT (http://sift.bii.a-star.edu.sg/) were used to predict the pathogenicity of non-synonymous variants. The candidate variants were confirmed by Sanger sequencing and familial segregation testing was performed whenever possible. The workflow is summarized in Figure 1.

Genotype data has been deposited at the European Genome-phenome Archive (EGA, http://www.ebi.ac.uk/ega/), which is hosted by the EBI, under accession number EGAS00001000683.

### Sanger Dideoxy Terminator Sequencing

Selected variants were confirmed by Sanger dideoxy terminator sequencing. Primers were designed with Primer3 software. PCR was carried out with HotStarTaq PCR kit (Qiagen Inc.,USA) and GC-Rich PCR kit (Roche Diagnostics GmbH, Mannheim, Germany) was used for GC rich regions. Sequencing products were resolved on an ABI3730 capillary sequencing instrument (Applied Biosystems) and chromatograms were analysed with Mutation Surveyor version 3.3 (SoftGenetics, LLC, State College, PA, USA).

### RNA extraction and cDNA sequencing

RNA was extracted from freshly collected whole blood using the NucleoSpin RNA blood kit (Machery-Nagel GmbH, Germany) and cDNA was synthesized using the Qiagen OneStep RT-PCR kit (Qiagen Inc., USA). PCR primers were designed within exons and PCR was performed using cDNA as template. PCR products were visualised on gels and sequenced as described above.

## Results

### Data output and quality

For all eight patients, 81–84% of the targeted regions achieved read depths of at least 20×, with average read depths overlapping targets ranging from 322× to 798× (Table 2). There were a few genes with very poor coverage. In particular, coverage of *IKBKG*, *CFC1* and *GTF21* was generally poor. In addition, read depths were <10× for a few exons in genes like *ARX*, *SHANK3*, *MECP2*, *SALL1*, *TBX1*, *PGA5*, *NF1*, *CHRNA7*, *NIPA1*, *DMD*. These were usually either in exon 1 or within GC rich regions.

**Table 2:** Summary of MPS data for the eight patients.

Sample	Read Pairs (M)	% Aligned to Reference	Reads overlapping target (%)	Ave Target Depth	Target ≥20× (%)	Total SNV	Variants in dbSNP	Novel variants
Patient 1	32.30	82.6	53.4	487	82	15482	14368	172
Patient 2	20.53	87.6	55.4	322	81	9699	9183	129
Patient 3	51.92	80.5	54.4	798	83	23885	21794	236
Patient 4	48.25	81.7	52.8	719	84	23716	21946	228
Patient 5	18.93	88.1	56.5	304	81	9204	8603	158
Patient 6	30.44	86.0	55.7	478	83	13726	12803	179
Patient 7	48.76	81.0	51.4	707	83	26268	23517	272
Patient 8	24.33	86.1	57.1	392	82	10598	9955	131

### Variant detection and prioritization

After filtering out the common variants present in dbSNP and variants with MAF >1% in EVS and the 1000 Genome browser, 1505 single nucleotide variants (SNVs) were identified in the eight patients, with a range of 131 to 236 SNVs per patient. A small number of variants were selected for validation by Sanger sequencing based on the prioritization criteria shown in Figure 1.

### Patient 1

**Figure 2:**
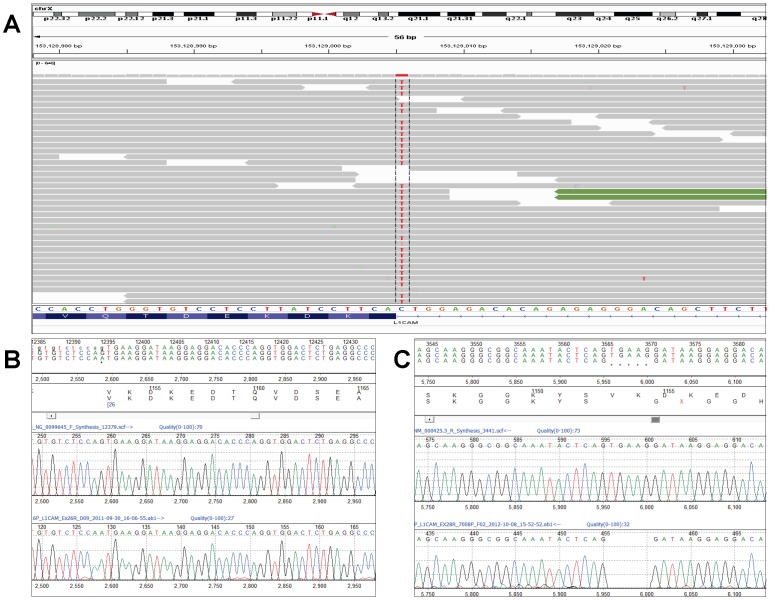
*L1CAM* splice site mutation in Patient 1. (A) IGV snapshot of c.3458-1G>A variant in the *L1CAM* gene (Chr X:153129005, hg19). (B) Sanger sequencing confirmation of c.3458-1G>A variant (NM_000425.3) (C) Partial cDNA sequence showing the mutant allele with the 5 bp deletion.

A total of 172 SNVs were detected in Patient 1 who is male with a 46,XY karyotype. Of the SNVs affecting exons, the top candidate mutation was a putative splice site mutation in the *L1CAM* gene, c.3458-1G>A, located at chrX: 153129005 (hg19) which was sequenced at 88× (Figure 2A). This variant was confirmed by Sanger sequencing (Figure 2B) and parental testing confirmed that the hemizygous mutation was inherited from his healthy mother. The variant affects the invariant AG acceptor splice site of intron 25 and is predicted to affect the splicing of exon 26. cDNA sequencing confirmed that splicing is affected at exon 26 and that the variant results in a deletion of 5 bp (r.3458_3462delTGAAG) from exon 26,which causes a frame shift and a premature stop leading to a predicted truncated protein of 1153 amino acids (Figure 2C).

### Patient 2

**Figure 3:**
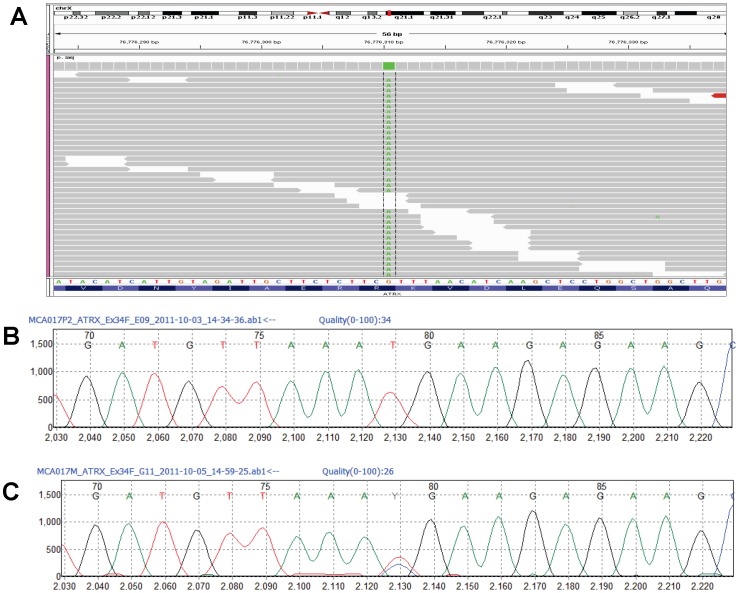
*ATRX* p.R2386X mutation in Patient 2. (A) IGV snapshot of c.7156C>T variant in *ATRX* (Chr X:76776310, hg19). (B) Sanger sequencing confirmation of c.7156C>T (p.R2386X, NM_00489.3) in Patient 2. (C) Sanger sequencing showing heterozygous c.7156C>T variant in the mother.

Patient 2 is male with a 46,XY karyotype. Among the 129 SNVs detected in this patient, the top candidate was a c.7156C>T (p.R2386X) mutation in the *ATRX* gene, located at chr X: 76776310 (Figure 3A). The mutation was validated by Sanger sequencing (Figure 3B), and extended family testing showed that the mutation was present in the unaffected mother and in an affected brother (Figure 3C), but was absent in the father and unaffected sister.

### Patient 3

**Figure 4:**
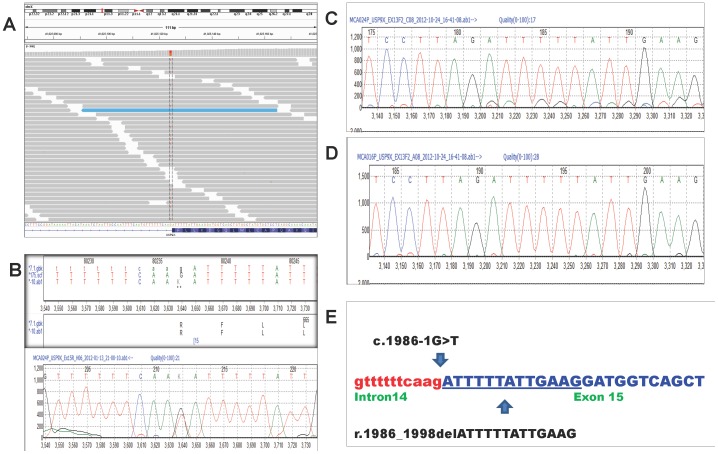
*USP9X* splice site mutation in Patient 3. (A) IGV snapshot of c.1986-1G>T variant in *USP9X* (Chr X:41025124, hg19). (B) Sanger sequencing confirmation of c.1986-1G>T variant (NM_001039590.2) in Patient 3. (C) Partial cDNA sequence showing expression of both the wild type and low level mutant allele with the 13 bp deletion. (D) Partial cDNA sequence of control patient. (E) Partial genomic DNA sequence of exon 15 (uppercase, blue) and intron 14 (lowercase, red) of *USP9X* gene showing the c.1986-1G>T variant (arrow) and the 13 bp deletion (r.1986_1998delATTTTTATTGAAG) which is underlined.

A total of 236 SNVs were detected in Patient 3, who is female with a 46,XX karyotype. The top candidate SNVs included potential splice site SNVs in *USP9X* and *FGFR2*, and non-synonymous variants in *RELN*, *DMD*, *MAP7D2* and *ZNF41*. Indels in exonic regions of *SHROOM4*, *ZIC2* and *MED12* were also investigated. All the SNVs and indels were confirmed by Sanger sequencing and parental testing showed that all the variants were inherited except for the *USP9X* splice site variant. The *de novo* c.1986-1G>T variant in *USP9X* (chrX: 41025124) is the likely causative mutation (Figure 4A & 4B). This variant is novel and is predicted to affect the acceptor splice site of intron 15 of the *USP9X* gene. cDNA sequencing with primers in exon 13 and exon 17 showed the normal transcript together with the low-level presence of an altered transcript. The altered transcript showed a deletion of 13 bases (r.1986_1998delATTTTTATTGAAG) which is predicted to result in an altered protein and a premature stop codon p.F663Mfs*18 (Figure 4C & 4E). A second, independent pair of primers in exons 13 and 16 also showed the presence of this low-level altered transcript. Sequencing of the RT-PCR product from a control RNA only showed the presence of the normal transcript with no altered transcript seen (Figure 4D)

### Patients 4–8

The top candidate variants of patients 4–8 are listed in Table 3. All these variants have been confirmed by Sanger sequencing and their inheritance was tested if parental samples were available. In addition to the variants detected by MPS, previous array CGH using Affymetrix SNP6 for Patient 5 showed a 134 kb deletion at 9p21.1 extending from 28,609,725–28,743,782 (hg19) involving the *LINGO2* gene (data not shown). This copy number change was initially thought to have no clinical significance.

**Table 3:** Assessed candidate variants found in patients 4–8.

Patient	Gene	Coordinates (hg19)	Nucleotide change	Amino acid change	Prediction		Inheritance
					PolyPhen-2	SIFT	
4	*CREBBP*	Chr16:3820816	G>A	p.P879S	Benign	Tolerated	Maternal
	*CTNNA3*	Chr10:67829183	T>C	p.K681R	Benign	Tolerated	Paternal
	*TSC1*	Chr9:135804244	T>C	p.N6D	Benign	Tolerated	Maternal
	*FMR1*	ChrX:147019073	A>G	p.E360G	Benign	Tolerated	Maternal
	*GPR50*	ChrX:150348482	C>T	p.R143C	Probably damaging	Deleterious	Maternal
5	*ANKRD11*	Chr16:89346082	C>T	p.P2290S	Benign	Tolerated	Maternal
	*CACNA1C*	Chr12:2800220	A>G	p.N2091S	Benign	Tolerated	Not maternal^a^
	*AHI1*	Chr6:135611614	C>A	p.D1179Y	Benign	Deleterious	Not maternal^a^
	*MAP7D3*	ChrX:135314112	G>A	p.T335M	Benign	Tolerated	Maternal
6	*NIPA1*	Chr15:23049158	G>A	p.P221S	Possibly damaging	Tolerated	Maternal
	*NIPA1*	Chr15:23049147	C>G	p.Q224H	Benign	Tolerated	Maternal
	*CTNND2*	Chr5:11411650	T>G	p.E146A	Possibly damaging	Tolerated	Paternal
7	*SYNGAP1*	Chr6:33411463	C>G	p.A1045G	Benign	Tolerated	Not tested
	*SEMA5A*	Chr5:9318508	C>A	p.Q82H	Benign	Deleterious	Not tested
	*AHI1*	Chr6:135644371	T>C	p.E1086G	Probably damaging	Deleterious	Not tested
	*MAP7D3*	ChrX:135310836	C>T	p.R611H	Probably damaging	Tolerated	Not tested
	*FLNA*	ChrX:153593613	C>T	p.V528M	Probably damaging	Deleterious	Not tested
8	*FOXP1*	Chr3:71102908	G>C	p.A100G	Probably damaging	Deleterious	Maternal
	*AUTS2*	Chr7:69755471	G>A	p.V260I	Unknown	Tolerated	Maternal
	*ATRX*	ChrX:76938923	G>C	p.P609A	Benign	Tolerated	Maternal
	*CNTN3*	Chr3:74420458	T>A	p.I183L	Benign	Tolerated	Maternal
	*CSMD1*	Chr8:2800085	C>T	p.G1374S	Unknown	Deleterious	Paternal
	*DMD*	ChrX:32364161	G>C	p.Q1829E	Benign	Tolerated	Maternal

a Father's DNA not available.

## Discussion

The average read depths achieved for the targeted regions were very high (>322×) and read depths of >20× were achieved for 81–84% of the targets. There was some variation in coverage, with overall poor coverage for just three genes and poor coverage in a few exons for another 10 genes.

Targeted MPS detected the causative mutations in *L1CAM* in Patient 1 and *ATRX* in Patient 2, which were consistent with their respective phenotypes. In Patient 1 the c.3458-1G>A mutation in the *L1CAM* gene affected the splicing process and cDNA sequencing confirmed the presence of a truncated transcript. Mutations in the *L1CAM* gene are associated with L1 syndrome, an X-linked recessive disorder. The major features of L1 syndrome include congenital hydrocephalus, adducted thumbs, spastic paraplegia, agenesis of the corpus callosum and cognitive impairment [6]. These features were all present in Patient 1. The c.3458-1G>A mutation in Patient 1 is novel but a splice site mutation at the same position (c.3458-1G>C) has been reported in a male with L1 syndrome [7].

In Patient 2, a p.R2386X mutation in the *ATRX* gene segregated with the clinical phenotypes in the family. Mutations in *ATRX* cause X-linked alpha thalassaemia mental retardation (ATR-X syndrome) in males, which is associated with profound developmental delay, facial dysmorphism, genital abnormalities and alpha thalassemia [8]. The features in Patient 2 were largely consistent with ATR-X syndrome. The p.R2386X mutation occurs in exon 34 of the ATRX protein. This mutation has been reported in three separate instances in the literature and all cases have involved severe mental retardation and genital abnormalities [9]. Independent testing of this family by conventional ATRX molecular testing corroborated our MPS result [10].

Patient 3 has multiple congenital anomalies. A *de novo* c.1986G>T mutation in the *USP9X* gene is likely to be causative for all or some of her clinical phenotypes. We showed the presence of a truncated cDNA sequence which is predicted to result in a truncated protein. It is possible that the truncated protein might have a dominant negative effect or impaired protein function. USP9X is a member of the USP family of deubiquinating enzymes (DUBs) which process ubiquitin precursors and ubiquinated proteins. It has an important regulatory role in protein turnover and is an essential component of the TGFβ/BMP signalling cascade through its control of SMAD4 monoubiquitination [11]. The drosophila homolog (FAF) is essential for normal eye development and embryonic viability and the mouse homolog (FAM) has been shown to play essential roles in embryonic development [12]–[14]. There has been one published report of a truncating mutation in *USP9X* which segregated with disease in an X-linked mental retardation family [15]. Identification of more patients with mutations in the *USP9X* gene will be needed to confirm the association with ID and congenital anomalies.

Patients 4, 5, 6, 7, 8 have phenotypes that included autism spectrum disorders. Autism spectrum disorders are complex disorders. Despite the compelling argument for a genetic basis for autism, it is estimated that a specific genetic cause has been established for only 15% of cases [16]. Recent large exome studies in trios have implicated a number of genes, but did not identify any gene as a major cause of autism [17]–[19]. The exome studies all showed the extreme heterogeneous nature of autism and also indicated that many of the genes implicated were interconnected by shared pathways. The exome trio studies also showed the importance of *de novo* point mutations in autism.

Single variants in a number of genes were detected but variants in *AHI1* and *MAP7D3* were seen in Patient 5 and Patient 7. *AHI1* is highly expressed in fetal brain and also in the cerebellum and cerebral cortex of the adult brain, and mutations in *AHI1* cause Joubert Syndrome [20]. Genetic association has also been reported between *AHI1* and autism [21]. The *AHI1* variant E1086G is a pathogenic mutation in a case of Joubert syndrome [22]. The E1086G and D1179Y variants have been reported in the EVS database with a frequency of 0.008% and 0.033% respectively. *MAP7D3* has not been associated with autism spectrum disorders. There was one report of a truncating variant in *MAP7D3* in an X-linked mental retardation family that did not segregate with disease. The T335M variant has been reported in the EVS database with a frequency of 0.019% (2 of 10444 alleles).

Novel variants in *CREBBP*, *TSC1* and *FMR1* were detected in Patient 4. These genes have been associated with syndromic autism and were probably unlikely to be causative of her phenotypes as they were inherited from her healthy parents. Similarly there were interesting candidate variants in Patient 6 in *NIPA1* gene which has been associated with autism. Again these variants were inherited and thus less likely to be causative of his autism spectrum disorder.

In Patient 7, variants were found in *SYNGAP1* and *SEMA5A* which have been linked to autism [23]. This patient also had a V528M variant in *FLNA* that has been reported in a case of bilateral periventricular nodular heterotopia [24] and also as a functional polymorphism [25]. Novel variants in *FOXP1*, *ATRX*, *AUTS2*, *CSMD1* were detected in Patient 8. All these genes have been associated with autism and other neurodevelopmental disorders. However, all these variants are inherited from her healthy parents and thus their role in her phenotype remains uncertain. In particular mutations and deletions in *FOXP1* have been shown to cause autism, mental retardation and speech and language deficits [26], [27]. The A100G variant in *FOXP1* has been predicted to be damaging by PolyPhen 2 and SIFT. The novel V260I variant in *AUTS2* is also of interest. This variant is present only in AUTS2 isoform 3, the shortest isoform comprised of 266 amino acids. The *AUTS2* gene was first identified as a candidate gene for autism when it was shown to be disrupted by a translocation breakpoint in a pair of autistic twins [28]. Mutations in the *AUTS2* gene have also been identified in patients with mental retardation [29].

Patient 5 also had a 134 kb deletion at 9p21.1 affecting the 5′UTR and first two exons of the *LINGO2* gene. This deletion was not present in his mother and the father's DNA was not available for testing. Deletions affecting the *LINGO2* gene have been reported in a cohort of Utah autism patients [30]. There is a possibility that the 9p21.1 deletion could act in conjunction with other SNVs in Patient 5 to influence his phenotype. Recent publications have highlighted the potential effects of polygenic mutational events, variable expressivity, variable penetrance, and CNV burden on complex disorders like autism [31]–[34]. All these factors complicate the diagnosis of the causes of autism spectrum disorders. The unavailability of some parental samples and the lack of complete phenotyping of parents in our study also limit the ability to predict the causality of some of the variants detected.

Despite the progress in genetic testing, 50–60% of the causes of DD/ID remain unknown. This is unfortunate as a clinical diagnosis of DD/ID provides crucial information for diagnostics and helps in understanding the mechanisms of the disease including the options for management and treatment. In addition, it puts an end to the testing odyssey and gives families closure and may also be important for future reproductive decisions.

This pilot study has confirmed the value of targeted MPS for investigating DD/ID and multiple congenital anomalies in children for diagnostic purposes. Causative mutations were found in two of the eight patients tested and a likely causal mutation was found in another patient. For the other five patients, no conclusions can be made about the variants as no compelling *de novo* variants were found. Thus, targeted gene MPS was less likely to provide a genetic diagnosis for children whose phenotype includes autism.

The advantages of targeted gene sequencing as opposed to whole exome sequencing is the increase in numbers of patients who could be sequenced and hence an increase in the number of patients who might receive a diagnosis. With targeted gene sequencing a greater depth of coverage could be achieved at a lower cost. The increased depth will facilitate the detection of indels that might be missed by exome sequencing. Targeted sequencing also obviates the problem of incidental results. Clinical interpretation of novel variants remains challenging but should gradually become easier with the continued development of variant databases of healthy controls as well as locus-specific disease databases.

## Supporting Information

Table S1 List of 355 genes in the targeted sequencing panel.
(XLS)
